# 24-Hour Movement Behavior Predict Working Memory Across Time In Older Adults: National Health And Aging Trends Study

**DOI:** 10.1093/geroni/igaf122.4266

**Published:** 2025-12-31

**Authors:** John Oginni, Rebecca Bergee, Jennifer Schrack, Zan Gao

**Affiliations:** University Of Tennessee-knoxville, Knoxville, Tennessee, United States; University of Tennessee-Knoxville, Knoxville, Tennessee, United States; Johns Hopkins Bloomberg School of Public Health, Baltimore, Maryland, United States; University of Tennessee-Knoxville, Knoxville, Tennessee, United States

## Abstract

Cross-sectional studies have identified associations between 24-hour movement behaviors — moderate-to-vigorous physical activity (MVPA), light physical activity (LPA), sedentary time (SED), and sleep — and cognitive outcomes. However, it remains unclear how these behaviors, measured at a single time point, predict older adults’ cognitive outcomes over time. This study investigated the longitudinal relationship between 24-hour movement behaviors and subsequent working memory in older adults, using three waves of follow-up data (2021–2023) from 492 U.S. Medicare beneficiaries aged 65 and older in the National Health and Aging Trends Study (NHATS). Structural equation modeling with an autoregressive cross-lagged panel design revealed that a 60-minute increase in SED at Time 1(T1) and Time 2(T2) predicted declines in working memory at T2 (B = -0.02, p < 0.001) and Time 3 (T3) (B = -0.013, p = 0.004), respectively. Conversely, a 15-minute increase in MVPA at T1 was associated with improved working memory (B = 0.013, p = 0.01) at T2, and increases in MVPA at T2 predicted better working memory at T3 (B = 0.019, p < 0.001). Similarly, a 60-minute increase in LPA at T1 and T2 predicted better working memory at T2 (B = 0.02, p < 0.01) and T3 (B = 0.03, p = 0.001), respectively. Notably, sleep duration at T1 was positively associated with working memory at T2 (B = 0.016, p = 0.008). These findings suggest that MVPA, LPA, and adequate sleep may support cognitive function over time, while prolonged sedentary behavior may be detrimental.

